# Diagnosis and Treatment of Pineal Region Tumors in Adults: A EURACAN Overview

**DOI:** 10.3390/cancers14153646

**Published:** 2022-07-27

**Authors:** Giuseppe Lombardi, Pietro Luigi Poliani, Renzo Manara, Moncef Berhouma, Giuseppe Minniti, Emeline Tabouret, Evangelia Razis, Giulia Cerretti, Vittorina Zagonel, Michael Weller, Ahmed Idbaih

**Affiliations:** 1 Department of Oncology, Oncology 1, Veneto Institute of Oncology IOV-IRCCS, Via Gattamelata 64, 35128 Padua, Italy; giulia.cerretti@iov.veneto.it (G.C.); vittorina.zagonel@iov.veneto.it (V.Z.); 2 Pathology Unit, Department of Molecular and Translational Medicine, University of Brescia, P.le Spedali Civili 1, 25121 Brescia, Italy; luigi.poliani@unibs.it; 3 Neuroradiology Unit, University of Padua, Via VIII Febbraio 2, 35122 Padua, Italy; renzo.manara@unipd.it; 4 Department of Neurosurgery, Lyon University Hospital, 69007 Lyon, France; moncef.berhouma@chu-lyon.fr; 5 Radiation Oncology Unit, Department of Medicine, Surgery and Neuroscience, University of Siena, Via Banchi di Sotto 55, 53100 Siena, Italy; giuseppe.minniti@unisi.it; 6 IRCCS NEUROMED, 86077 Pozzilli (IS), Italy; 7 Neuro-Oncologye Department, Aix-Marseille University, La Timone Hospital, AP-HM, Team 8 GlioME, CNRS 7051, Inst. Neurophysiopathol, 13005 Marseille, France; emeline.tabouret@ap-hm.fr; 8 Third Department of Medical Oncology, Hygeia Hospital, 15123 Athens, Greece; erazis@hygeia.gr; 9 Department of Neurology, University Hospital and University of Zurich, 8006 Zurich, Switzerland; michael.weller@usz.ch; 10 Sorbonne Université, Inserm, CNRS, UMR S 1127, Institut du Cerveau, ICM, AP-HP, Hôpitaux Universitaires La Pitié Salpêtrière—Charles Foix, Service de Neurologie 2-Mazarin, 75013 Paris, France; ahmed.idbaih@aphp.fr

**Keywords:** pineal region tumors, rare tumors, EURACAN, pinealoblastoma

## Abstract

**Simple Summary:**

Pineal region tumors are rare intracranial tumors. A deeper knowledge of these tumors’ molecular mechanisms has been gained in recent years, which has led to a new classification and new potential systemic treatments. Surgery remains the mainstay of treatment, while radiotherapy and systemic therapy depend on histological, molecular, and clinical characteristics. This paper highlights recent developments in the diagnosis and treatment of these tumors.

**Abstract:**

Pineal region tumors are rare intracranial tumors, accounting for less than 1% of all adult intracranial tumor lesions. These lesions represent a histologically heterogeneous group of tumors. Among these tumors, pineal parenchymal tumors and germ cell tumors (GCT) represent the most frequent types of lesions. According to the new WHO 2021 classification, pineal parenchymal tumors include five distinct histotypes: pineocytoma (PC), pineal parenchymal tumors of intermediate differentiation (PPTID), papillary tumor of the pineal region (PTPR), pinealoblastoma (PB), and desmoplastic myxoid tumor of the pineal region, SMARCB1-mutant; GCTs include germinoma, embryonal carcinoma, yolk sac tumor, choriocarcinoma, teratoma, mixed GCTs. Neuroradiological assessment has a pivotal role in the diagnostic work-up, surgical planning, and follow-up of patients with pineal masses. Surgery can represent the mainstay of treatment, ranging from biopsy to gross total resection, yet pineal region tumors associated with obstructive hydrocephalus may be surgically managed via ventricular internal shunt or endoscopic third ventriculostomy. Radiotherapy remains an essential component of the multidisciplinary treatment approach for most pineal region tumors; however, treatment volumes depend on the histological subtypes, grading, extent of disease, and the combination with chemotherapy. For localized germinoma, the current standard of care is chemotherapy followed by reduced-dose whole ventricular irradiation plus a boost to the primary tumor. For pinealoblastoma patients, postoperative radiation has been associated with higher overall survival. For the other pineal tumors, the role of radiotherapy remains poorly studied and it is usually reserved for aggressive (grade 3) or recurrent tumors. The use of systemic treatments mainly depends on histology and prognostic factors such as residual disease and metastases. For pinealoblastoma patients, chemotherapy protocols are based on various alkylating or platinum-based agents, vincristine, etoposide, cyclophosphamide and are used in association with radiotherapy. About GCTs, their chemosensitivity is well known and is based on cisplatin or carboplatin and may include etoposide, cyclophosphamide, or ifosfamide prior to irradiation. Similar regimens containing platinum derivatives are also used for non-germinomatous GCTs with very encouraging results. However, due to a greater understanding of the biology of the disease’s various molecular subtypes, new agents based on targeted therapy are expected in the future. On behalf of the EURACAN domain 10 group, we reviewed the most important and recent developments in histopathological characteristics, neuro-radiological assessments, and treatments for pineal region tumors.

## 1. Introduction

EURACAN is the European Reference Network for all rare adult solid cancers (http://euracan.eu, accessed on 26 July 2022). This network involves healthcare providers and patient representatives across Europe with the aim of tackling rare adult solid cancers. Among EURACAN networks, there is a subgroup of highly specialized physicians for rare cancer of “the brain and spinal cord” (called “Domain 10”). On behalf of the EURACAN Domani 10 group, we performed this overview on regional pineal tumors. 

**Figure 1:**
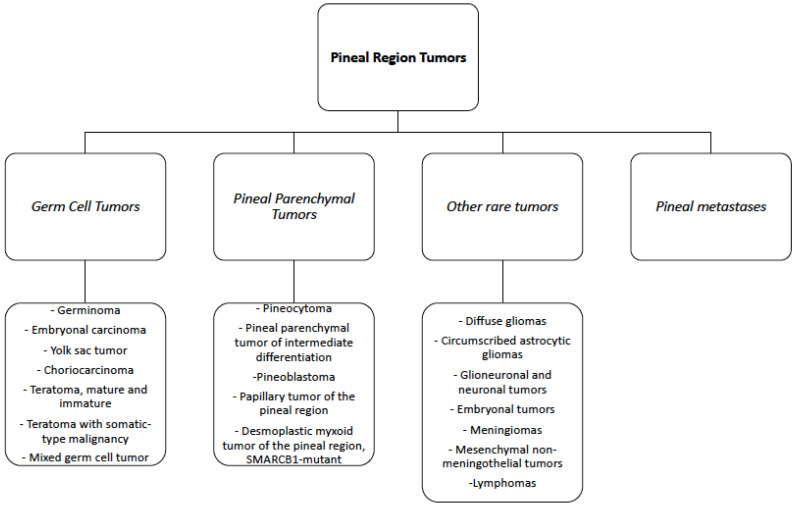
Summary of the Pineal Region Tumors according to the WHO 2021 classification.

The pineal region is a composite anatomical space comprising the pineal gland and surrounding structures [1], including the epithalamus, quadrigeminal cistern, and posterior wall of the third ventricle [2]. Tumors of the pineal region (PRT) are rare, accounting for less than 1% of all intracranial tumor lesions, and represent a histologically heterogeneous group of tumors, including primary pineal parenchymal tumors, germ cell tumors (GCT), and tumors originating from the adjacent structures, such as choroid plexus tumors, meningiomas, and gliomas [3]. Lymphomas, atypical teratoid rhabdoid tumor (ATRT), and metastases may also occur in this region, though they are uncommon (see Figure 1) [4,5,6]. Pineal parenchymal tumors and germ cell tumors together make up more than 70% of all pineal region tumors [7]. Treatment options range from biopsy and surgery to radiotherapy and chemotherapy. However, treatment is not standardized due to the low incidence of these tumors, the different histological types, and the small number of studies reported in the literature. 

On behalf of the EURACAN domain 10 group, we reviewed the most important and recent developments in histopathological characteristics, neuro-radiological assessments, and treatments for pineal region tumors. 

## 2. Pathological and Molecular Features 

Approximately 30% of all pineal region tumors are of pineal parenchymal origin, and according to the World Health Organization (WHO) classification, pineal parenchymal tumors include four distinct histotypes stratified according to histological features and grade: pineocytomas (PC; WHO Grade 1), pineal parenchymal tumors of intermediate differentiation (PPTID; WHO Grades 2 and 3), papillary tumor of the pineal region (PTPR; WHO grade 2 and 3), and pinealoblastoma (PB; WHO Grade 4). The majority of PBs occur in children, whereas PCs and PPTIDs are more common in young adults. In addition, the most recent WHO 2021 classification of central nervous system (CNS) tumors [8] recognizes a new entity, the Desmoplastic Myxoid Tumor (DMT) of the pineal region, based on the SMARCB1 mutation and lacking histological markers of malignancy (see Table 1). 

PC represents approximately 25% of all primary parenchymal pineal tumors. PCs are usually well-circumscribed, slow-growing tumors with a favorable prognosis, composed of well-differentiated cells resembling mature pineocytes within a glioneuronal stroma and forming expansive lobules. The tumors are non-infiltrative with rare mitotic figures, no necrosis, and minimal nuclear atypia. Features of neuronal differentiation (ganglion cells) may be present. A distinct feature is the presence of pinocytomatous rosettes composed of neoplastic cells surrounding a central neuropil core. Tumor cells are diffusely immunoreactive for synaptophysin and neurofilament. The proliferative rate, assayed by the percentage of tumor cells labeled for Ki-67/Mib1, is low (an average of 1 to 2%). Molecular studies on PCs are few and mainly restricted to cytogenetic alterations involving chromosomes X, 5, 8, 11, 14, 19, and 22 [9]. 

PPTIDs present a challenging diagnosis due to the absence of stringent histopathologic criteria for their classification and the presence of histological features spanning from well-differentiated PCs to high-grade PBs [7,10]. PPTID accounts for nearly 40–45% of all primary parenchymal pineal tumors. PPTIDs have a heterogeneous architecture devoid of the primitive small round cell appearance seen in PBs, and endothelial hyperplasia or necrosis is rarely detected, depending on grade. WHO Grade 2 PPTIDs generally retain a higher expression of neurofilament, similar to PCs, with a low mitotic count (<5 mitoses per 10 HPF) but with a Ki67/MIB-1 labeling index generally higher than in PCs, ranging from 6% to 10%. WHO Grade 3 PPTIDs have a diffuse growth pattern devoid of PC-like regions, loss or very limited neurofilament expression, and a progressive acquisition of an undifferentiated phenotype. Accordingly, the mitotic count is higher (>5 mitoses per 10 HPF) with a Ki67/MIB-1 labeling index ranging from 10 to 20%. Endothelial proliferation and necrotic foci are also commonly found. Unlike PCs, PPTIDs show an increased rate of molecular alterations, particularly for WHO Grade 3 PPTIDs. Overexpression of genes such as PRAME, CD24, POU4F2, and HOXD13 has recently been reported in PPTID grade 3, representing a potential useful biomarker in the differential diagnosis with PPTID grade 2. Moreover, higher expression of CD24 and PRAME may also have a prognostic value, as it is associated with significantly shorter survival [11]. Chromosome 4q and 12q gains, as well as chromosome 22 loss, have been reported as frequent chromosomal alterations in PPTID [12]. Recent advances in both genomic and transcriptomic profiling have enabled the characterization of oncogenic drivers. The KBTBD4 in-frame insertion is a frequent molecular alteration in PPTID. In contrast, DICER1 and DROSHA mutations were limited to PBs and may aid in differential diagnosis [7,13]. ATRX mutations associated with protein loss have also been reported [14]. 

PBs account for approximately 30% of all parenchymal pineal tumors and about 10% of all pineal region tumors; they occur primarily in children or infants. PBs are composed of poorly differentiated and immature cells that display rapid growth and a predilection for leptomeningeal dissemination [15]. Histologically, PBs show marked hypercellularity composed of small round immature cells with hyperchromatic nuclei frequently showing anaplasia with frequent mitotic figures, apoptotic bodies, nuclear molding, necrosis, and a high Ki67/Mib1 labeling index ranging from 20–25% and up to 50% or more. Homer–Wright rosettes may also be present in tumors. Synaptophysin expression is mostly retained, while neurofilaments are usually absent and GFAP may be partially positive. Diagnosis, especially in small biopsies, is challenging, and it may be difficult to distinguish PBs from other malignant tumors occurring in this region, including GCTs, ATRTs, high-grade gliomas, and WHO grade 3 PPTIDs [16]. PBs frequently show high expression levels of several molecules, including UBEC2, SOX4, TERT, TEP1, PRAME, CD24, POU4F2, and HOXD13 [17,18]. The expression levels of CRX, a master transcriptional regulator of photoreceptor differentiation expressed in the pineal gland and retina, are useful to support their pineal lineage [19,20]. DNA methylation profiles have revealed four clinically relevant and biologically different PB subgroups with different age at diagnosis, metastatic propensity, molecular alterations, and clinical outcomes [21,22,23]. Recurrent homozygous deletions, as well as mutations of the microRNA-processing pathway genes (DICER1, DROSHA, and DGCR8), identify PB subtypes designated as microRNA-processing altered group 1 and microRNA-processing altered group 2, both frequently occurring in older children (age 3–18 years), with an intermediate to favorable prognosis (5-year OS ranging between 70 to 100%) [13,24]. Notably, PB microRNA-processing altered groups are the most prevalent CNS malignancy in DICER1 predisposition syndrome patients carrying germline DICER1 mutations [25,26]. FOXR2 overexpression and MYC activation characterize the more aggressive MYC/FOXR2-altered PB subgroup, which occurs mainly in infants and young children. [22]. Loss of function of the RB1 gene characterizes the PB RB1-altered subgroup originating mainly in infants (median age 1–2 years) with dismal prognosis and rapid progression (5-year OS less than 30%) [23]. Cytogenetic alterations are also encountered, with frequent gain on chromosomes 1, 6, 7, 12, 17 and loss on chromosomes 8, 14, 16. Interestingly, chromosome 16q loss has been associated with a significantly worse prognosis in MYC/FOXR2-altered and RB1-altered PBs [22]. Thus, methylation profiling reveals a considerable molecular heterogeneity within different PBs associated with different clinical features and survival. In this regard, molecular sub-grouping is of paramount importance to guide patient stratification for future clinical studies and personalized treatment. 

The DMT SMARCB1-mutant of the pineal region is a newly recognized rare entity that primarily affects young adults and exhibits distinct clinical and histopathological features [8]. Histologically, the DMT SMARCB1-mutant is composed of small to medium-sized cells within a loose basophilic myxoid matrix with irregularly shaped elongated blood vessels. Focal calcifications may be present, mitotic activity is low, and tumor necrosis is absent. All cases exhibited loss of nuclear SMARCB1/INI1 protein expression and were immunoreactive for EMA and CD34 [27]. The Ki67/MIB1 proliferation index is low in the majority of cases (median 3%). Interestingly, the DMT SMARCB1-mutant of the pineal region shares a close methylation profile with ATRT-MYC, a recently recognized ATRT methylation molecular subgroup carrying SMARCB1 deletion and endowed with a relatively good prognosis [28]. 

PTPR is a rare WHO grade 2 or 3 pineal tumor characterized by variable morphology, an epithelial appearance, a predominantly papillary architecture reminiscent of ependymomas, and the presence of ependymal rosettes [29]. Despite its anatomical association with the pineal gland, a PTPR is believed to originate from specialized cells in the posterior third ventricle near the pineal gland that show ultrastructural features of ependymal and neuroendocrine differentiation [30]. PTPR usually expresses cytokeratins (including CK18) along with S100, Vimentin, and neuron-specific enolase (NSE), but not neurofilaments, whereas expression of epithelial membrane antigen (EMA) and GFAP is inconsistent [29]. Mitotic figures are uncommon, although necrosis is often present. Increased mitotic (≥3 mitoses per 10 HPFs) and proliferative activities (Ki67/MIB1 index ≥ 10%), along with loose papillary structures, have been shown to be associated with shorter progression-free survival [31]. Nearly all PTPR cases exhibit chromosome 10 loss associated with mutations in the pten gene and activation of the PI3K pathway [1]. Additional alterations are frequent losses of chromosomes 3 and 22q, as well as gains of chromosomes 8p, 8, and 12 [30]. Nuclear FOXJ1 expression, commonly expressed in normal ependymal cells and ependymal neoplasms, allows for discrimination with histopathological mimics, particularly PPTID [30]. 

Finally, germ cell tumors (GCTs) represent up to 50–60%of pineal tumors in the pineal region [32]. GCTs are more frequent in young adults and children, predominantly in male patients. Germinomas are the most common type of pineal tumor, but all the different histotypes may be present, including choriocarcinomas, teratomas, embryonal carcinomas, yolk sac tumors, and mixed germ cell tumors [3]. Other tumors, including metastatic cancer spreading to the pineal gland, are exceedingly uncommon [4,5,6].

## 3. Neuroradiological Assessment

Neuroimaging plays a pivotal role in the diagnostic work-up, surgical planning, and follow-up of patients with pineal masses. Both computerized tomography (CT) and magnetic resonance imaging (MRI) can provide useful information regarding the location, size, and shape of the pineal tumor. While MRI remains the preferred tool for tumor characterization, brain CT is often the first-line imaging method for patients presenting with rapid neurological deterioration or progressively worsening headaches. CT is sufficient for detecting pineal region tumors or hydrocephalus, secondary to compression of the tectum of the midbrain and obstruction of the aqueduct. In addition, CT is preferable for the assessment of calcifications and hemorrhage. MRI best reveals lesion enhancement patterns, the presence of concomitant lesions in the suprasellar region, or the presence of leptomeningeal dissemination. In addition, MRI better differentiates neoplasms from other benign pineal region masses such as pineal, epidermoid, dermoid, or arachnoid cysts.

In general, neuroradiological literature clearly typifies most types of pineal region tumors, delineating their main morphological and signal or density features (see below). However, the high number of cell types and the variety of brain structures in this area, as well as the common overlap of neuroimaging features among different tumor types, make the differential diagnosis rather challenging. In most cases, the definitive diagnosis may be determined by clinical data (age and sex) and imaging results, but ultimately relies on laboratory serum or cerebrospinal fluid tumor markers and histological and immunohistochemical examinations. 

Even though neuroimaging is often inconclusive with regard to the nature of the lesion, it is crucial in the management of pineal mass lesions by differentiating benign conditions from neoplasms, narrowing the differential diagnosis of pineal region neoplasms, defining the relationship with vascular (e.g., internal cerebral veins) and parenchymal structures of the pineal region, and detecting leptomeningeal or regional spread or the coexistence of other lesions. In addition, neuroimaging has become an indispensable tool for post-surgical lesion follow-up, which is usually scheduled according to the histological lesion’s definitive characterization. CT is typically used in the very early post-surgical period to exclude surgery-related complications and to determine the severity of hydrocephalus. However, subsequent neuroimaging evaluation is usually performed with contrast-enhanced MRI, often including the whole neuraxis. 

The following is a neuroradiological description of the three major categories of pineal region tumoral masses: parenchymal cell tumors, germ cell tumors, and neoplasms of the supporting tissues (glial tumors). Metastasis, vascular, and benign lesions are also reported.

### 3.1. Pineal Parenchymal Tumors

− Pineocytoma generally appears as a well-circumscribed tumor with a maximum dimension of less than 3 cm. Compared to the adjacent brain, the lesion appears iso-/hyperdense on CT, hypo-/isointense on T1, and iso-hyperintense on T2. The tumor tends to be solid and presents strong homogeneous enhancement. Cystic changes may be present which, in some cases, can make it difficult to differentiate it from a pineal cyst; a nodular or thickened wall enhancement helps identify its neoplastic nature [33]. Intralesional hemorrhage may occur, while calcifications are usually peripheral, distinguishing pineocytomas from pineal germinomas that tend to engulf pineal calcification. Pineal apoplexy is reported [34].
− Pineoblastomas usually present as large (more than 3 cm), irregular, poorly defined lobulated tumors, often invading the adjacent brain. On CT, they appear hyperdense compared to the adjacent brain due to high cellularity, with frequent necrotic areas and hemorrhagic changes due to their highly malignant nature. As in pineocytomas, calcifications are peripherally dispersed (“blasted calcifications”). A cystic appearance may occur, but the walls are usually thicker and more irregular than in pineal cysts. On MRI, pineoblastomas present heterogeneous signal intensity (hypo- to isointense on T1 and iso- to hyperintense T2) and restricted diffusion. Besides elevated choline and decreased N-acetylaspartate, MR spectroscopy reveals slightly elevated glutamate and taurine peaks (∼3.4 ppm). Contrast enhancement is vivid and CSF seeding is present in 15% of patients at the time of diagnosis and up to 45% during the course of the disease. Consequently, screening of the entire neural axis is necessary both at the time of biopsy diagnosis/imaging suspicion and during follow-up. Obstructive hydrocephalus is common at presentation.
− Pineal parenchymal tumors of intermediate differentiation range from well-circumscribed pineal tumors with pineocytoma-like characteristics to poorly defined, invasive masses with rapid growth and/or low ADC values. They often appear iso-hyperintense on T2, and may present cystic areas and heterogeneous contrast enhancement [34]. As these tumors may spread along the CSF, contrast-enhanced MRI of the entire craniospinal axis is required.
− Papillary tumors of the pineal region also appear on MRI as well-circumscribed T2-hyperintense masses with variable contrast enhancement. Intralesional cysts are common, and their proteinaceous content may result in a heterogeneous T1 signal. Contrast-enhanced MRI of the entire craniospinal axis is recommended because local tumor recurrence and leptomeningeal tumor spread may occur.

### 3.2. Germ Cell Tumors

Tumors of germ cell origin include germinomas, teratomas, and, less commonly, embryonal carcinoma, pineal yolk sac tumor, and choriocarcinoma.

− On CT, a pineal germinoma typically appears as a homogeneous, hyperdense mass compared to the adjacent brain. Cysts are present in 20–52% of cases [33,35,36]. Inner calcifications are common and often represent normal pineal calcifications engulfed within the tumor. On MRI, germinomas are usually T1 and T2 isointense to grey matter, DWI comparatively hyperintense, and ADC values are typically higher than in pineoblastomas. Contrast enhancement is vivid. Disseminated disease and subependymal tumor spread along the third ventricle are common at onset (13%).
− Teratomas often manifest as large, multiloculated, lobulated lesions containing cysts and solid components, as well as intralesional areas of fat, calcifications, and fluid lesions [37]. On CT, the majority of teratomas demonstrate at least some highly hypodense or hyperdense components (fat and calcification, respectively). On MRI, teratomas may present T1-hyperintense components due to fat and proteinaceous/lipid-rich fluid and T1-hypointense components due to calcification and blood products. Given the extremely variable histological components, T2-weighted imaging also tends to be heterogeneous, with hypo- or hyperintense intratumoral components. After contrast medium administration, solid components show variable enhancement. Immature teratomas appear more homogeneous, with irregular infiltrating margins and with fewer cysts and calcifications, thus becoming more difficult to differentiate from other pineal tumors. Secondary somatic malignancies are not rare; therefore, surveillance for both secondary malignancy and growing teratoma syndrome is recommended [37]. Mature teratomas are well-circumscribed and often present fat components that help narrow the differential.
− Pineal yolk sac tumors are rare and do not seem to differ from other germ cell tumors. These lesions are usually large, irregular, and frequently infiltrate the adjacent structures; they present variable densities and calcifications. On MRI, the lesions are hypo-isointense on T1 and hyperintense on T2-weighted images. Contrast enhancement is intense and homogeneous [38].
− Embryonal carcinomas usually have large masses that are iso-hypointense on T1 and iso-hyperintense compared to grey matter on T2 [39]. Their margins are lobulated and irregular; invasion of the adjacent structures is suggested by edema along the tumor margins. Cystic areas are common; the solid portions show vivid contrast enhancement.
− Pineal choriocarcinomas are large [40], highly vascular [41] lesions with a propensity to hemorrhage (as do their metastases). The masses are usually ovoid and relatively well-defined, although irregular infiltrating margins have been observed. Most lesions are iso-hypointense compared to the cortex on T1 and markedly heterogeneous on T2. Contrast enhancement is usually marked but heterogeneous. Micro and macrocysts or necrotic areas and mild-to-moderate peritumoral edema are common. Hemorrhages result in signal heterogeneity with blooming on T2* and SWI and with hyperintense foci on T1. On CT, the tumor appears heterogeneously hypodense; calcifications are uncommon.
− Radiologic characteristics and survival outcomes of most frequent pineal parechymal tumors and germ cell tumors are summarized in Table 2.

### 3.3. Other Neoplasms of the Pineal Region

The variety of brain structures in this area results in a high number of possible neoplasm types (e.g., meningiomas, gliomas, melanomas, ependymomas, etc.). In addition, the pineal region may also harbor metastases.

− Pineal meningiomas are well-circumscribed masses, mostly arising from the contiguous tentorium, the tela choroidea, or the velum interpositum [53]. On CT, lesions are often iso- or hyperdense compared to grey matter; calcifications are detected in 15–20% of cases [34]. The MRI signal is variable. The mass shows intense homogeneous contrast enhancement, typically involving the contiguous dural structures (dural tail).
− Primary pineal melanoma is exceedingly rare and can present as either melanotic or amelanotic MRI patterns. The former is more common, and the mass appears hyperintense on T1 and hypo- or isointense on T2 due to the presence of melanin. The amelanotic MRI pattern occurs less frequently, and the lesion is hypo- or isointense on T1 and hyperintense on T2 (less than 10% of cells contain melanin). Contrast enhancement is typically present [34].
− Ependymomas appear heterogeneous, usually hypo- to isointense on T1 and iso- to hyperintense on T2. Cysts, calcifications, and hemorrhages are common. Contrast enhancement may be present; diffusion restriction is heterogeneous, but the ADC is predominantly high due to relatively low cellularity.
− Pineal gliomas include pilocytic astrocytoma, IDH-mutant astrocytoma and oligodendroglioma, glioblastoma, and choroid plexus papilloma [54], although well-differentiated astrocytomas are the most prevalent [55]. Apart from tumors originating from the pineal glands, gliomas of the pineal region also include tectal, thalamic, and splenial gliomas. Imaging features are therefore rather heterogeneous based on the tumor’s grading and anatomical origin. Intralesional necrosis, calcifications, cysts, and hemorrhages are variably found; contrast enhancement is also variable.
− Pineal metastases should be considered in patients with a history of any malignancy. Although lung cancer is the most frequently implicated, pineal metastases of almost all malignant tumors have been reported [56]. On neuroimaging, metastases are often isodense/isointense to grey matter. Lesion margins may be well-demarcated or may infiltrate the adjacent structures. Vivid contrast enhancement is the rule; necrosis and cysts may be present. Leptomeningeal seeding has been observed in two-thirds of cases; contrast-enhanced MRI of the entire craniospinal axis is therefore required.

### 3.4. Non-Neoplastic and Benign Pineal Region Lesions

Several conditions may be observed in this complex region.

− Pineal cysts are very common in the general population (up to 40% in autopsy series) and almost always asymptomatic. On MRI, benign pineal cysts are usually oval, thin-walled, uni- or multi-loculated, or filled with a proteinaceous or hemorrhagic fluid that usually does not restrict on DWI. On MRI, the cysts are hypo- to isointense on T1 and iso- to hyperintense on T2 and FLAIR compared to grey matter. Common features are a thin rim of calcification (25% of cases) and, above all, a smooth (i.e., non-nodular) thin rim of wall enhancement, usually <2 mm in thickness. Internal enhancement may be observed on delayed images due to seepage of gadolinium into the cyst fluid. There is no invasion of adjacent structures, and minimal or no mass effect. The midbrain aqueduct remains unobstructed [57].
− Cysts of the velum interpositum are a common anatomic variation with dilatation of the normal cistern of the velum interpositum (diameter > 1 cm). The cyst usually assumes a triangular configuration pointing anteriorly and typically incorporates the internal cerebral veins along its lateral walls. On CT and MRI, the cyst’s content has the features of CSF.
− Arachnoid cysts appear as a sharply defined extra-axial fluid collection, which is similar in signal to CSF on all MRI sequences, including DWI. There is no post-contrast enhancement. When severe hydrocephalus is present, arachnoid cysts of the pineal region should be differentiated from ventricular diverticula, as the latter usually disappear with ventricle shunting.
− Epidermoid cysts are non-neoplastic lesions that mostly appear hypodense on CT; focal calcifications may be observed. On MRI, these masses usually present CSF-like signals on T1 and T2, but exhibit a bright signal on DWI [58]. Contrast enhancement is typically absent, although faint, very late peripheral enhancement may be observed.
− Dermoid cysts are usually well-defined rounded midline masses that commonly appear markedly hypodense on CT, hyperintense on T1, and hypointense on fat-saturated T1 due to intracyst lipid content [59]. Calcification may be present in the wall. Typically, dermoid cysts do not enhance after contrast administration, although a thin peripheral rim may be observed. The cyst’s rupture may release lipid droplets into the subarachnoid spaces, aiding in the distinction between lipomas and mature teratomas.
− Pineal lipomas are easily recognized with CT due to the very low density of fat. Calcifications may be present. On MRI, the well-demarcated lesion appears homogeneously hyperintense on T1 and hypointense on fat-suppressed imaging. The typical lack of contrast enhancement helps to differentiate lipomas from mature teratomas.
− Vein of Galen aneurysm malformation is the abnormal dilation of the embryonic precursor to the vein of Galen due to congenital arteriovenous malformations. It is usually observed in neonatal or fetal imaging. This condition is easily identified by MR-angiography and signal void due to blood flow.
− Cavernomas are rare, but usually their MRI characteristics allow a prompt diagnosis in the majority of cases. On T2 images, they usually have a “pop-corn like” appearance surrounded by a hypointense hemosiderin ring. MRI signal intensity depends on whether there has been recent hemorrhage or thrombosis [60].

## 4. The Role of Surgery 

Advanced microsurgical techniques (endoscopy, neuronavigation, electrophysiological monitoring) associated with considerable improvements in anesthetic and resuscitation management have facilitated the surgical management of PRTs. Except for germ-cell tumors and lymphomas, a maximal microsurgical removal remains the gold-standard for PRTs. 

### 4.1. Management of Hydrocephalus

More than half of PRT cases are associated with obstructive hydrocephalus at the time of diagnosis [61]. In this case, the management of hydrocephalus should be promptly discussed, and ventricular internal shunt or, preferably, endoscopic third ventriculostomy (ETV) can be utilized. The latter is preferable since, in addition to the relief from hydrocephalus, it provides the opportunity to perform a biopsy when the tumor bulges in the posterior part of the third ventricle with a sensitivity greater than 90% and less than 3% of significant complications, mainly due to intraventricular hemorrhage in highly vascularized tumors. A classical ventricular internal shunt (ventriculoperitoneal or ventriculoatrial) also carries the risk of neoplastic dissemination. A CSF sample is systematically collected for the PRT markers’ analysis and neoplastic cell screening. During follow-up, approximately 15% of patients who have been treated with ETV may require an internal ventricular shunt because of third ventricle stoma dysfunction [61,62]. 

### 4.2. Biopsy

**Figure 2:**
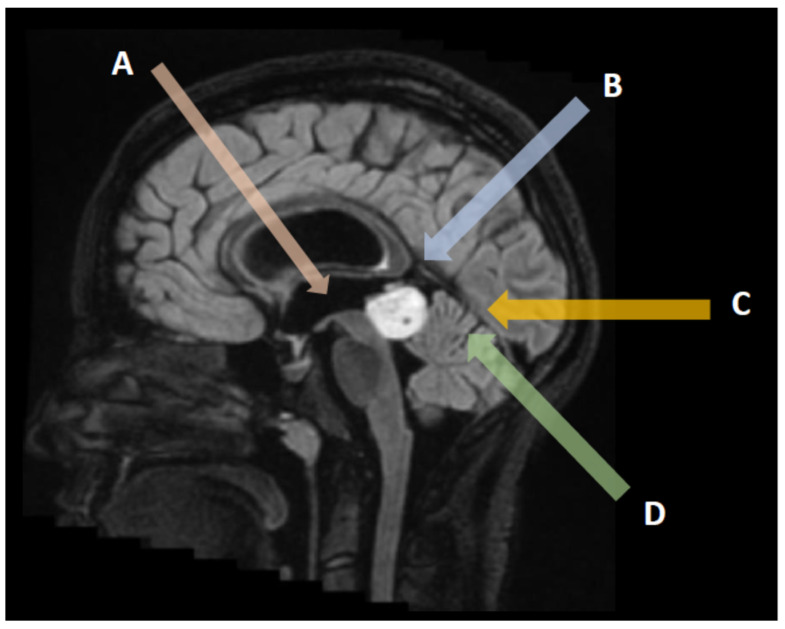
Surgical approaches to pineal region tumors: Endoscopic transventricular third ventriculostomy and biopsy with CSF sampling (**A**), interhemispheric parietal approach (**B**), suboccipital transtentorial approach (**C**), and supracerebellar infratentorial approach (**D**).

Tissue sampling is of paramount importance prior to multidisciplinary therapeutic discussion. In the majority of patients presenting with hydrocephalus, a biopsy is possible during the third ventriculostomy itself, particularly in large tumors extending anteriorly within the third ventricle cavity (Figure 2A) [63]. In the other patients, a stereotactic biopsy is usually performed under neuronavigation, providing a histological diagnosis in 87–97% of cases (Figure 2B–D). The drawback of biopsies in PRT remains the risk of obtaining a non-representative sample in mixed tumors containing different tumoral contingents. Despite the proximity of venous complex anatomy (Galen vein and tributaries), the morbidity and mortality of PRT biopsies remain similar to those of other encephalic locations (mortality 0–2%, transient morbidity 7–8.4%, and definitive morbidity inferior to 1.2%) [64].

### 4.3. Surgical Removal

The surgical excision of PRT remains the standard when blood and/or CSF markers are negative and should always be discussed in a multidisciplinary meeting involving a neuro-oncologist, a radiotherapy specialist, and a neurosurgeon. Because of their depth and critical venous relationships, PRTs should be surgically managed in tertiary centers having extensive experience with these tumors. In large series, surgical mortality is less than 3%, but morbidity may reach 20%, particularly in cases with oculomotor and visual field abnormalities. The choice of a specific surgical approach depends upon the tumor extensions in relation to the Galen venous complex and the surgeon’s experience. 

The suboccipital transtentorial (Figure 2C) approach is preferable for tumors extending upward and displacing the venous complex inferiorly. The patient is usually placed in a sitting position in the absence of an open ovale foramen, otherwise in a three-quarter prone position (Park Bench). The craniotomy should expose both lateral and superior sagittal sinuses on the right side in a right-handed patient. The occipital lobe is then gently dissected and retracted to expose the falcotentorial dural angle and the straight sinus. The tentorial dura is then incised one cm parallel to the straight sinus until reaching the thick arachnoid layers of the pineal region. Care should be taken to preserve all the veins tributaries of the Galen complex during dissection and tumor debulking. The tumor is separated from the superior aspect of the cerebellum and the tectal plate until the lumen of the third ventricle is entered. Laterally the fourth nerve may be encountered and preserved. This approach carries a significant risk of visual field dysfunction because of the occipital lobe retraction, usually transient but unfortunately sometimes permanent. 

The infratentorial supracebellar approach (Figure 2D) provides a straightforward route to the pineal region, particularly for tumors developing inferiorly to the venous complex. For an optimal exposure, the patient is placed in a sitting position when possible, to let the cerebellum move down naturally with gravity. Sometimes it is necessary to sacrifice one or two bridging veins between the superior surface of the cerebellum and the tentorium. The approach can be slightly lateralized on a paramedian axis to avoid injuring the midline bridging veins. This approach provides a direct route to the pineal region inferior to the Galen venous complex. The main potential complication is cerebellar injury due to a venous infarct or contusion during retraction, as well as a tectal plate dysfunction (oculomotor disturbances) and pneumocephalus. 

Other approaches are possible, depending on the tumor’s extension laterally or in the third ventricle [65,66,67,68]. A significant lateral extension may be managed by a transcortical transparietal approach with a high risk of visual field complications. A more anterior extension within the third ventricle can be approached through a transventricular approach (transforaminal, interforniceal…) with significant risk of memory disturbances.

In a recent retrospective study, Shepard et al. analyzed the surgical and oncologic outcomes of 68 patients who underwent surgery for a pineal region tumor [69]. The mean age at surgery was 30.9 ± 15.3 years, pre-operative hydrocephalus was present in 83.8% of cases, the median pre-operative Karnofsky Performance Status (KPS) was 90. Germ cell tumors were 20.6% of cases and pineal parenchymal tumors were 30.9% of cases. Glial tumors comprised 32.4% of lesions. In this study, gross total resection (GTR) was achieved in 52.9% of patients and lower grade tumors were associated with improved rates of resection. About adverse events, 45.6% of patients had one or more adverse events within 30 days of surgery; among these, the most common adverse events were new or worsening hydrocephalus (14.7%) and post-operative focal motor deficits (10.3%). The 30-day mortality was 5.9%, mostly due to intratumoral hemorrhage (three out of four cases). Gross total resection, low grade tumors and improved performance status were associated with improved overall survival. Due to the small number of cases, no analysis was performed to assess the impact of histopathology on overall survival [69]. 

### 4.4. Specific Management of Pineal Cysts

Pineal cysts are common benign expansive cysts of the pineal gland, found in up to 5% of brain imaging [64]. 

The majority are inferior to 10 mm and remain asymptomatic, but rarely they can expand and cause aqueduct stenosis with subsequent hydrocephalus. They may also be revealed by headaches, oculomotor disturbances by compression of the tectal plate or cerebellar symptoms. Benign pineal cysts usually appear as hypoT1, hyperT2 cystic lesion with a slightly enhancing thin wall. A thicker wall and/or associated solid component should be considered as neoplastic and managed as a cystic pineal region tumor. Asymptomatic benign pineal cysts should be annually followed to ensure the absence of evolution. The management of symptomatic ones remains controversial. One commonly used option in patients with benign pineal cysts causing symptomatic hydrocephalus is an endoscopic third ventriculostomy allowing at the same time the possibility to biopsy the cyst wall and fenestrate it within the third ventricle lumen. A microsurgical direct approach can also be proposed in symptomatic patients when hydrocephalus is absent (oculomotor or cerebellar signs) [64].

## 5. The Role of Radiotherapy

Radiotherapy remains an essential component of the multidisciplinary treatment approach for intracranial germ cell tumors. Significant advances in radiotherapy techniques have been made in recent decades, resulting in a more precise and effective treatment. Current advanced external-beam radiation techniques include image-guided radiotherapy (IGRT), intensity-modulated radiotherapy (IMRT), stereotactic radiosurgery (SRS), and volumetric modulated arc therapy (VMAT) [70]. Modern techniques provide highly conformal dose distributions with improved target volume coverage and sparing of normal tissues compared to three-dimensional conformal radiotherapy techniques, with the potential to reduce the risk of long-term sequelae, in particular neurocognitive dysfunction. In addition, there is increasing interest in particle therapy with protons; for large intracranial lesions, its dosimetric advantage over photons provides a more favorable dose distribution to the surrounding normal tissue. Treatment volumes include craniospinal irradiation, whole-brain irradiation, whole ventricular irradiation, or focal radiotherapy. These volumes depend on the histological subtypes, grading, extent of disease, and the combination with chemotherapy.

Current radiotherapy indications for different tumor types are summarized in Table 3. For patients with germinoma, craniospinal irradiation with 36 Gy followed by a boost to 50–54 Gy for the primary tumor, using fractions of 1.8 Gy per fraction, was considered the standard treatment until the 1990s, with a reported 5-year event-free survival of more than 90% [49,50]. Given the overall excellent prognosis for localized germinomas and concern about the long-tern toxicity of radiotherapy, including second malignancies, strokes, and neurocognitive decline [71,72], clinical management has shifted to chemoradiation, which includes lower radiation doses and smaller target volumes [73]. For localized germinoma, the current standard of care is chemotherapy followed by reduced dose whole ventricular irradiation using 24 Gy, plus a boost to the primary tumor of 16 Gy in 1.8 Gy per fraction [73], with lower doses of 18 Gy and 12 Gy given to patients with complete response after chemotherapy, as recommended by the Children’s Oncology Group [74]. 

Similarly, bifocal germinomas may also be treated with whole ventricular irradiation and primary boost. The use of whole ventricular irradiation is currently recommended because of an excess of recurrences in the ventricles with the use of involved field radiotherapy [73]. When radiotherapy is used as a single modality approach, treatment fields include whole ventricle radiotherapy to a dose of 24 Gy and tumor boost to a dose of 40–45 Gy (1.8 Gy per fraction). For patients with disseminated disease, treatment requires craniospinal irradiation of 30.4–36 Gy in 18–20 daily fractions, with a boost to primary and metastatic sites of 40–45 Gy in 1.8 Gy per fraction [73]; however, craniospinal and tumor boost doses can be reduced in patients receiving combined chemoradiation [75]. 

The non-germinomatous germ cell tumor group comprises several histologies, including embryonal carcinoma, endodermal sinus tumors (yolk sac tumors), choriocarcinoma, immature teratoma, teratoma with malignant transformation, and mixed tumors. In comparison to pure germinomas, these tumors are less radiosensitive and their prognosis following radiotherapy alone is poor (5-year survival of 20% to 45%) [51]. Therefore, the goal is to obtain a complete response before radiotherapy. Following 4–6 cycles of platinum-based chemotherapy, craniospinal irradiation (30–36 Gy) plus tumor boost (50.4–54 Gy total) is the standard of care in North America [76,77], while focal radiotherapy for localized tumors and craniospinal irradiation for metastatic disease only is more frequently used in Europe based on the SIOP-CNS-GCT-96 trial [78].

The majority of evidence on the role of radiotherapy for pineal tumors is derived from retrospective studies and small case series, making it challenging to define a standard treatment. Pineoblastoma occurs more frequently in pediatric patients than in adult patients [79]. In the pediatric population, postoperative radiation has been associated with higher overall survival [80,81,82]; in contrast, there is a lack of high-quality data on the impact of radiotherapy in adult pineoblastoma. In an analysis of 213 adult patients with pineoblastoma collected by the Surveillance, Epidemiology, and End Results (SEER) database from 1975 to 2016, postoperative radiotherapy (hazard ratio 0.43, *p* < 0.05) and combined postoperative chemoradiation (hazard ratio 0.38, *p* < 0.05) emerged as independent prognostic factors for survival [83]. In a comprehensive review of 109 studies that collectively described the outcomes of 299 patients with pineoblastoma, Tate et al. [45] found an overall survival rate of 54% at a mean follow-up of 31 ± 1.9 months (range, 1–159 months). The addition of adjuvant radiotherapy was not associated with improved survival following complete resection, although subtotal resection followed by radiotherapy resulted in improved survival; 2-year survival rates were 53% following subtotal resection and 64% after subtotal resection followed by adjuvant radiotherapy (*p* < 0.05). Although the efficacy of radiotherapy is still debated, adjuvant cranial–spinal irradiation (24–38 Gy) to the entire axis and (45–54 Gy) to the tumor in 1.8–2.0 Gy fractions combined with platinum-based systemic chemotherapy is currently recommended following maximal surgical resection.

For other pineal tumors, including pineal parenchymal tumor of intermediate differentiation, papillary tumor of the pineal region, and desmoplastic myxoid tumor, SMARCB1-mutant, clinical behavior, and histological grading criteria are yet to be defined, and the role of adjuvant postoperative therapy remains unclear. Currently, indications for radiotherapy are mainly based on small retrospective studies and it is usually reserved for aggressive (grade 3) or recurrent tumors, while observation is indicated for tumors showing less aggressive behavior, e.g., grade 1–2 tumors. Pineal parenchymal tumor of intermediate differentiation, grade 2 or 3 according to the WHO Classification of CNS Tumors, is a rare tumor arising from the pineal parenchyma that occurs more commonly in adults with limited aggressiveness, lying between the spectrum of pineocytoma and pineoblastoma (WHO 2021). Although many centers recommend adjuvant radiation, the optimum treatment for these tumors remains undefined. In an individual patient analysis of 29 studies that involved 127 patients, Mallick et al. [46] reported better survival for 46 patients who received either craniospinal radiation or local radiation than those who did not (252 vs. 168 months; *p* = 0.009), with no difference according to the different radiation approaches. While the management of these tumors still remains unclear due to lack of evidence, adjuvant radiotherapy is usually recommended in all patients with pineal parenchymal tumors of intermediate differentiation who underwent partial or subtotal resection. Local radiation doses of 50.4–54 Gy using modern conformal techniques should be preferred to limit long-term treatment-associated toxicity. Craniospinal irradiation is usually recommended for grade 3 tumors.

As for other pineal tumors, the treatment of papillary tumors of the pineal region is not well-defined, and no standard guidelines are available. In a recent review of 177 patients with papillary tumors of the pineal region covered in 77 articles, Yamaki et al. [48] observed a survival rate of 83.5% at 3 years, with a 56.8% recurrence rate after a median of 29 months. Surgical resection was associated with increased survival, although the extent of resection did not affect outcomes. Adjuvant treatments, including radiotherapy (44%), chemotherapy (10.3%), and radiosurgery (10.8%), did not improve survival. After multivariate analysis, tumor size and surgical treatment were associated with survival. Complete surgery, when feasible, is the recommended treatment for pineocytoma, with no requirement for further adjuvant therapies. In a review of the literature involving 64 articles and a total of 166 patients, Clark et al. found no significant difference in progression-free survival for subtotal resection only versus subtotal resection in addition to radiotherapy [42]. Five-year survival rates were around 86%, with local recurrence and even cerebrospinal fluid metastases, which are rarely reported. A few studies have evaluated a second course of radiotherapy for relapsing tumors of the pineal region, although its role in terms of efficacy and risk of toxicity remain controversial [84,85]. 

**Table 3:** Radiation therapy for tumors of the pineal region.

Tumor Type	WHO Grade	Treatment Strategy	Extent of Irradiation and Dose Fractionation
Pure germinoma [73,74]	NA	RT in combination with ChT or as an exclusive treatment	Limited disease: WVI 24 Gy and tumor boost 16 Gy; WVI 18 Gy and boost 12 Gy in 1.6–1.8 Gy fractions for patients with complete response; metastatic disease: CSI alone 24 Gy and tumor boost 16 Gy in 1.8 Gy fractions;
Non-germinomatous germ cell tumor [76,77,78]	NA	Postoperative RT in combination with ChT	Limited disease: focal RT 54 Gy or CSI 30.4–36 Gy and tumor boost 18–23.4 Gy; Metastatic disease: CSI, 30.4–36 Gy and tumor boost 18–24 Gy in 1.8 Gy fractions;
Pineoblastoma [80,81]	4	Postoperative RT in combination with ChT	CSI 24–36 Gy and tumor boost 45–54 Gy in 1.8–2 Gy fractions
Pineal parenchymal tumor of intermediate differentiation [46]	2,3	Postoperative RT alone or in combination with ChT	Focal RT 50.4–54 Gy or CSI 24–36 Gy and tumor boost 45–54 Gy in 1.8–2 Gy fractions;
Papillary tumor of the pineal region [48]	2,3	Incompletely resected or recurrent tumors	Focal RT 50.4–54 Gy in 1.8–2 Gy fractions;
Pineocytoma [42]	1	Commonly not indicated. To be evaluated in recurrent inoperable tumors	Focal RT 50.4–54 Gy in 1.8–2 Gy fractions or SRS 15–18 Gy (small residual or recurrent tumors)

RT, radiation therapy; ChT, chemotherapy; WVI, whole ventricular irradiation; CSI, craniospinal irradiation; NA = not available.

## 6. The Role of Sytemic Treatment

Chemotherapy is mainly used in the first-line treatment of pineal tumors, depending on the histology and prognostic factors such as residual disease and metastases. In this paragraph, we will specifically review chemotherapy approaches for pinealoblastoma and germ cell tumors (see Table 4). For other indications, chemotherapy protocols are similar to those proposed for other localizations.

### 6.1. Pinealoblastoma

Chemotherapy protocols for pinealoblastoma in adult patients are mainly derived from pediatric clinical trials. Various chemotherapy protocols are used in association with radiotherapy with a 5-years PFS rate of 60–70% for non-metastatic patients. The use of chemotherapy alone is not recommended and is associated with very poor prognosis [81]. Chemotherapy protocols are based on various alkylating or platinum agents, vincristine (VCR), etoposide, and cytarabine combinations. In the CCG-921 trial [86], the use of a randomized chemotherapy, either prednisone, lomustine (CCNU) and VCR, or “eight-drugs-in-one-day” (methylprednisolone, VCR, CCNU, procarbazine, hydroxyurea, cisplatin, cytarabine, and cyclophosphamide) following craniospinal irradiation was associated with a 73% 3-year OS rate. In the SIOP PNET 3 study [87], a 71% 5-year OS was reported with an alternance of VCR, VP16, and carboplatin or cyclophosphamide plus irradiation. In the HIT91 trial [80], five out of six children older than 3 years treated with sandwich or adjuvant chemotherapy with irradiation were alive and disease-free at the end of the study with a median OS of 8.8 years. In the HIT2000 trial [88] evaluating irradiation followed by adjuvant chemotherapy based on lomustine, cisplatin, and VCR, patients achieved a 5-year OS rate of 64%. Finally, a 5-year OS rate of 81% was achieved in the COG 99701 study [89] evaluating the concomitant use of VCR and carboplatin during irradiation followed by adjuvant cyclophosphamide and a VCR +/− cisplatin regimen. 

In parallel, up-front regimens with high-dose myeloablative chemotherapy (HDC) followed by autologous hematopoietic stem cell rescue (ASCR) have been developed with interesting control rates. In 2003, Gururangan and colleagues reported a 4-year OS rate of 71% with an HDC regimen composed of melphalan and cyclophosphamide or busulfan followed by ASCR [90]. In 2009, Chintagumpala and colleagues reported a 5-year OS rate of 67% after an HDC regimen composed of cisplatin, VCR, and cyclophosphamide followed by ASCR [91]. Finally, the SJMB03 trial [26] evaluated a risk-adapted radiotherapy regimen followed by HDC including cisplatin, VCR, and cyclophosphamide followed by ASCR. This study reported a 5-year OS rate of 100% in the intermediate-risk group and 60% in the high-risk group (metastasis and/or bulky residual disease). 

At relapse, HDC with ASCR could be an interesting strategy [92,93]. An HDC regimen including carboplatin, etoposide, and thiotepa following surgical debulking and preceding re-irradiation has also been proposed [94].

### 6.2. Germ Cell Tumor

Like its systemic counterpart, the chemosensitivity of pineal GCT is well known. Regular protocols are based on cisplatin or carboplatin and may include etoposide, cyclophosphamide, or ifosfamide.

Pure germinomas are very sensitive tumors with an excellent prognosis. The treatment of localized pure germinoma is composed of platinum-containing chemotherapy followed by irradiation. In the case of CNS metastases, pure germinomas are curable with craniospinal radiation without the need for additional chemotherapy. In the SIOP CNS GCT 96 study [73], 65 patients with germinoma were treated with carboplatin and VP16 alternating with ifosfamide and VP16 (“PE/IE” protocol) prior to irradiation. This protocol was associated with a 5-year PFS rate of 88% and a 5-year OS rate of 96%. In 2021, Bartels and colleagues reported the results of a COG study evaluating the combination of carboplatin and VP16 prior to a reduced irradiation protocol in the case of a complete response post-chemotherapy [95]. While this trial failed to demonstrate the non-inferiority of this approach, it was associated with an interesting chemotherapy response rate. Finally, the SIOP CNS GCT II trial evaluated a “PE/IE” combination for non-metastatic germinoma, followed by adapted irradiation based on response quality. This very promising trial is now closed for inclusion, and results are pending.

A regimen containing a platinum derivative is also the platform of systemic treatment for non-germinomatous germ cell tumors (GCT). The ACNS0122 [76] and ACNS1123 [77] protocols evaluated a regimen of carboplatin and etoposide alternating with ifosfamide and etoposide with very encouraging results, showing up to a 5-year PFS rate of 84% and a 5-year OS rate of 93%. The SIOP CNS GCT 96 trial [78] included 149 nongerminomatous tumor patients who were treated with cisplatin, etoposide, and ifosfamide chemotherapy (PEI) followed by irradiation. This protocol was associated with 5-year OS rates of 82% and 75% for localized and metastatic patients, respectively. Finally, the SIO CNS GCT II study evaluated two regimens of chemotherapy, including PEI HDC with ASCR, according to patient risk. The results are still pending, but this protocol could become the new first-line treatment for nongerminomatous GCT patients.

At relapse, GCT remains chemosensitive [85]. Thiotepa-based or melphalan-based HDC regimens followed by ASCR could be proposed [96,97,98,99]. For nongerminomatous GCT, the GEMPOX protocol, composed of gemcitabine, paclitaxel, and oxaliplatin, was associated with interesting responses in three patients [100].

**Table 4:** Clinical trial summary.

Pinealoblastoma						
Author	Date	Phase	Trial Name	Patients	Chemotherapy	Results
Liu [26]	2020	III	SJMB03	30 and 12 non-protocol	Risk-adapted IR + HDC (cisplatin/cyclphosphamide/VCR) + ASCR	IR: 5y-PFS & 5y-OS: 100% HR: 5y-PFS: 56%; 5y-OS: 60%
Gerber [88]	2014	II	HIT2000	11	IR + weekly concomitant VCR + adjuvant (lomustine/cisplatin/VCR)	5y-PFS & 5y-OS: 64%
Cohen [86]	1995	III	CCG-921	17	A: IR + weekly concomitant vincristine + adjuvant (VCR/CCNU/prednisone) B: neoadjuvant 8-in-1 (methylprednisolone/VCR/CCNU/procarbazine/hydroxyurea/cisplatin/cytarabine/cyclophosphamide) + IR + adjuvant 8-in-1	3y-PFS: 61%; 3y-OS: 73%
Gururangan [90]	2003	II		12	IR + HDC (cyclophosphamide/melphalan or busulfan/melphalan) + ASCR	4y-PFS: 69%; 4y-OS: 71%
Hinkes [80]	2007	IIB	HIT91	6	A: sandwich (ifosfamide/VP16/MTX/cisplatin/cytarabin) + IR + concomitant VCR +/− maintenance (carboplatin/VCR/CCNU) B: IR + concomitant VCR + adjuvant (CCNU/Cisplatin/VCR)	5y-PFS & 5y-OS: 83%
Pizer [87]	2006	III	SIOP PNET 3	10	Alternance (VCR/VP16/Carboplatin) and VCR/VP16/cyclophosphamide) + IR	5y-PFS & 5y-OS: 71%
Jakacki [89]	2015	I/II	COG 99701	23	IR + concomitant VCR and carboplatin + adjuvant (cyclophosphamide/VCR+/−cisplatin)	5y-PFS: 62%; 5y-OS: 81%
Massimino [101]	2006	II		3	MTX/VCR/VP16/cyclophosphamide/carboplatin + IR + maintenance (VCR/CCNU) or HDC (thiotepa) + ASCR	CR at the end of trial: 3/3
Chintagumpala [91]	2009	II		7	+/−topotecan + IR + HDC (cisplatin/cyclophosphamide/VCR) + ASCR	5y-PFS: 54%; 5y-OS: 67%
**Germ Cell Tumor**						
**Authors**	**Date**	**Phase**	**Trial Name**	**Patients**	**Chemotherapy**	**Results**
Calaminus [73]	2013	II	SIOP-CNS-GCT-96	65 G	carboplatin/VP16 alternating with ifosfamide/VP16 + IR	5y-PFS: 88%; 5y-OS: 96%
Allen [102]	1994	II		11 G	carboplatin + IR	91% in remission for a median of 25 months
Bartels [95]	2021	II		137 G	carboplatin/VP16 + IR	3y-PFS: 94%
Kretschmar [103]	2007	II	POG9530	12 G and 14 NG	cisplatin/VP16 alternating with VCR/cyclophosphamide + IR	G: 66mo-PFS: 92%; NG: 58mo-PFS: 79%
Da Silva [104]	2010	II	3rd international CNS GCT	25 (G and NG)	carboplatin/VP16 alternating with cyclophosphamide/VP16 alone	6y-PFS: 46%; 6y-OS: 75%
Fangusaro [77]	2019	II	ACNS1123	107 NG	carboplatin/VP16 alternating with ifosfamide/VP16 + IR	3y-PFS: 88%; 3y-OS: 92%
Calaminus [78]	2017	II	SIOP-CNS-GCT-96	149 NG	cisplatin/VP16/ifosfamide + IR	localized: 5y-PFS: 72%; 5y-OS: 82% metastatic: 5y-PFS: 68%; 5y-OS: 75%
Goldman [76]	2015	II	ACNS0122	102 NG	carboplatin/VP16 alternating ifosfamide/VP16 + IR	5y-PFS: 84%; 5y-OS: 93%

IR: irradiation; HDC: high-dose chemotherapy; ASCR: autologous hematopoietic stem cell rescue; VCR: vincristine; y: years; mo: months; PFS: progression-free survival; OS: overall survival; IR: intermediate risk; HR: high risk; G: germinomatous; NG: nongerminomatous.

## 7. The Future of Systemic Therapy

Although very little clinical research is currently taking place in regard to the treatment of pineoblastoma or pineal parenchymal tumors of intermediate differentiation, there is an increasing understanding of the biology of the disease’s various molecular subtypes [23]. This in-depth investigation into the characteristic mutations of these tumors is expected to lead to the development of targeted therapies. In the meantime, the optimization of chemotherapy regimens in use is being studied in the pediatric setting, and may lead to improvements in outcomes and side effect profiles [88].

## Figures and Tables

**Table 1:** Histological and molecular characteristics of pineal region tumors. PC = pineocytomas; PPTID = pineal parenchymal tumors of intermediate differentiation; PB = pinealoblastoma; DMT = desmoplastic myxoid tumor; PTPR = papillary tumor of the pineal region, TBD: to be defined.

Histotypes	WHO Grade	Frequent Age at Diagnosis	Mitotic Count (mitosis/10HPF)	Ki67/Mib1 LI (%)	Most Frequent Molecular Alterations
**PC**	1	adult	<0–1	1–2	n.s.
**PPTID**	2	adult	<5	6–10	KBTBD4 (insertion); ATRX loss of function
	3		5	10–20	
**PB (subtypes)**					
**PB-miRNA1**	4	older children or young adult	High mitotic count	20–25% and up to 50 or more	DICER1, DROSHA, DGCR8 (loss of function)
**PB-miRNA2**					DICER1, DROSHA (loss of function)
**PB-MYC/FOXR2**		Infant or young children			FOXR2 overexpression; MYC activation
**PB-RB1**					RB1 loss of function
**DMT-SMARCB1-mut**	TBD	young adult	<0–1	3	SMARCB1 loss of function
**PTPR**	2	young adult	<2–3	2–3	PTEN mutation; PI3K alteration
	3		≥3	≥10	

**Table 2:** Summary of radiologic characteristics and survival outcomes in most frequent pineal parechymal tumors and germ cell tumors.

Histotype	Radiological Characteristics	PFS	OS
**Pineal parenchymal tumors**			
**Pineocytoma**	- CT: iso/hyperdense - MRI: hypo/isointense on T1, iso/hyperintense on T2 - Well-circumscribed, solid, possible cystic changes - < than 3 cm ø - Strong homogeneous enhancement - Peripheral calcifications	5ys PFS 97% [42]	5 ys OS > 85% [42,43,44]
**Pineoblastoma**	- CT: hyperdense - MRI: hypo/isointense on T1 and iso/hyperintense T2, restricted diffusion; - Spectroscopy: choline, glutamate, taurine ↑, N-acetylaspartate ↓ - Irregular, lobulated, invasive tumor - Usually > than 3 cm ø - Strong contrast enhancement - Frequent necrosis and hemorrhage - Peripheral calcifications - 15% of pts with CSF seeding at diagnosis—45% in the course of the disease → neural axis screening necessary	-	5ys OS < 60% [43,44,45]
**Pineal parenchymal tumors of intermediate differentiation**	- Well-defined to invasive masses - MRI: iso/hyperintense on T2 - Possible cystic areas, heterogeneous contrast enhancement - Neural axis screening required	5ys PFS 74.1% [12]	5 ys OS 84.1% [46]
**Papillary tumors**	- Well-circumscribed - Variable contrast enhancement - MRI: hyperintense on T2, heterogeneous on T1 - Neural axis screening required	PFS 5ys 34.5% [47]	5ys OS < 75% [44,48]
**Germ cell tumors**			
**Germinoma**	- CT: homogeneous, hyperdense - MRI: isointense on T1 and T2, hyperintense DWI, higher ADC than pinealoblastoma - Cysts - Inner calcifications - Strong contrast enhancement - Neural axis screening required	5ys PFS 91% [49]	10ys OS 90% [50] 5ys OS > 90% [43,44,49]
**Teratoma**	- CT: both highly hypodense and hyperdense components present - MRI: hyper/hypointense on T1, hyper/hypointense on T2 - Large, multiloculated - Cysts and solid components are present - Variable contrast enhancement	5 ys PFS 20–45% [51]	5ys OS 30–100% [52] 5ys OS for immature teratoma: 30–70% [43] 5ys OS for mature teratoma: 90–100% [43]
**Pineal yolk sac tumors**	- MRI: hypo/isointense on T1, hyperintense on T2 - Intense and homogeneous contrast enhancement - Large, irregular, frequently infiltrating	-	5ys OS 60–70% [43]
**Embryonal carcinoma**	- MRI: iso/hypointense on T1, iso/hyperintense on T2 - Large, lobulated, invasive of surrounding structures (oedema) - Cystic areas	-	5ys OS 60–70% [43]
**Pineal choriocarcinoma**	- CT: heterogeneously hypodense - MRI: iso/hypointense on T1, heterogeneous on T2 - Strong but heterogeneous contrast enhancement - Large - Presence of hemorrhages, cysts, necrotic areas - Calcifications uncommon	-	5ys OS 45–70% [43]
